# Increased Vascularity in Cervicovaginal Mucosa with *Schistosoma haematobium* Infection

**DOI:** 10.1371/journal.pntd.0001170

**Published:** 2011-06-07

**Authors:** Peter Mark Jourdan, Borghild Roald, Gabriele Poggensee, Svein Gunnar Gundersen, Eyrun Floerecke Kjetland

**Affiliations:** 1 Centre for Imported and Tropical Diseases, Department of Infectious Diseases, Oslo University Hospital Ulleval, Oslo, Norway; 2 Faculty of Medicine, University of Oslo, Norway; 3 Centre for Paediatric and Pregnancy Related Pathology, Oslo University Hospital Ulleval, Oslo, Norway; 4 Department of Infectious Disease Epidemiology, Robert Koch Institute, Berlin, Germany; 5 Research Unit, Sorlandet Hospital HF, Kristiansand, Norway; 6 Centre for Development Studies, University of Agder, Kristiansand, Norway; Case Western Reserve University School of Medicine, United States of America

## Abstract

**Background:**

Close to 800 million people in the world are at risk of schistosomiasis, 85 per cent of whom live in Africa. Recent studies have indicated that female genital schistosomiasis might increase the risk of human immunodeficiency virus (HIV) infection. The aim of this study is to quantify and analyse the characteristics of the vasculature surrounding *Schistosoma haematobium* ova in the female genital mucosa.

**Methodology/Principal Findings:**

Cervicovaginal biopsies with *S. haematobium* ova (n = 20) and control biopsies (n = 69) were stained with immunohistochemical blood vessel markers CD31 and von Willebrand Factor (vWF), which stain endothelial cells in capillary buds and established blood vessels respectively. Haematoxylin and eosin (HE) were applied for histopathological assessment. The tissue surrounding *S. haematobium* ova had a higher density of established blood vessels stained by vWF compared to healthy controls (*p* = 0.017). Immunostain to CD31 identified significantly more granulation tissue surrounding viable compared to calcified ova (*p* = 0.032), and a tendency to neovascularisation in the tissue surrounding viable ova compared to healthy cervical mucosa (*p* = 0.052).

**Conclusions/Significance:**

In this study female genital mucosa with *S. haematobium* ova was significantly more vascularised compared to healthy cervical tissue. Viable parasite ova were associated with granulation tissue rich in sprouting blood vessels. Although the findings of blood vessel proliferation in this study may be a step to better understand the implications of *S. haematobium* infection, further studies are needed to explore the biological, clinical and epidemiological features of female genital schistosomiasis and its possible influence on HIV susceptibility.

## Author Summary

Schistosomiasis is a fresh water parasite infection that affects millions of people, especially in Africa. Recent knowledge about the genital manifestations of schistosomiasis; especially its possible association with human immunodeficiency virus (HIV) infection, has led to increased focus on this neglected tropical disease. Millions of women remain undiagnosed for genital schistosomiasis, and may suffer from abnormal mucosal blood vessels, contact bleeding and lesions named sandy patches. This study analyses a unique selection of female genital biopsies containing parasite eggs. Protein detection and standard histopathological assessment are combined to quantify and study the characteristics of the mucosal blood vessels surrounding the eggs. Our results show that the genital mucosa with parasite eggs is more vascularised compared to healthy tissue, and that viable eggs tend to be surrounded by proliferating blood vessels. These findings have not yet been correlated directly to clinical manifestations. Further studies are needed in order to provide clinical advice on the risks and consequences of mucosal lesions particular to female genital schistosomiasis.

## Introduction

Schistosomiasis is the most important parasite infection in the world after malaria [1]. Of the estimated 780 million people exposed to this fresh water parasite, 85 per cent live in Africa. *Schistosoma haematobium* and human immunodeficiency virus (HIV) are co-endemic in large parts of this area [2], [3]. In a recent cross-sectional study from Zimbabwe a near 3-fold increased odds for HIV infection was found in women with genital schistosomiasis [4].

The lower female reproductive tract is a major entry site in HIV transmission, and is also a common site for *S. haematobium* oviposition [5]–[7]. The *S. haematobium* infected cervix appears inflamed with abnormal mucosal blood vessels, contact bleeding and pathognomonic lesions named sandy patches [7]–[9]. It has been suggested that products from schistosome ova (*S. mansoni*) may induce endothelial cell proliferation and activation [10], [11]. Similar to sexually transmitted infections, it has been postulated that female genital schistosomiasis may provide mucosal points of entry for HIV [12], [13].

The aim of this study is to combine immunohistochemical protein detection of endothelial cells with histopathological evaluation to quantify and analyse the characteristics of the blood vessels surrounding *S. haematobium* ova in biopsies of the lower female genital tract.

## Methods

### Ethics statement

Permissions for the histopathological and immunohistochemical investigations of anonymised archival Malawian and Norwegian biopsies, without additional consent from the study subjects, were granted by the National Health Science and Research Committee of Malawi (2009/NHSRC #620) and the Norwegian Regional Ethics Committee (2009/1250a). The permissions were based on the fact that the proposed analyses did not require identifiable information or history, have any direct relevance to the physical, mental or social well-being, or have any direct diagnostic or therapeutic implications for the study women.

The majority of the Malawian women who volunteered in the 1994 study were illiterate [8]. Study information was provided in Yao and Chichewa (the local languages) and free informed oral consent was obtained. Following consent, all women who had urinary schistosomiasis were offered gynaecological examination. Consent was reascertained orally by the physician before each step of sampling and investigations. Treatment and follow-up for sexually transmitted infections, cancers and other complaints were done in collaboration with the physicians in Mangochi District Hospital [8]. The women were not asked for HIV testing. All women, including those who declined further investigations, were offered treatment with praziquantel. All non-endemic controls were followed up by the referring clinician in Norway.

### Study subjects

As described previously [8], biopsies from the cervix and/or vagina were sampled from 61 women with urogenital *S. haematobium* infection in Mangochi District Hospital in Malawi in 1994. In short, sexually active women between 15 and 49 years of age present in the out-patient department, irrespective of whether they were patients or next of kin, were invited to submit urine samples. Women with *S. haematobium* ova in the urine were invited for further interviews and gynaecological examinations. Samples including biopsies were taken from observed lesions or at random if no lesions were present. Women without urinary schistosomiasis were excluded.

**Figure 1:**
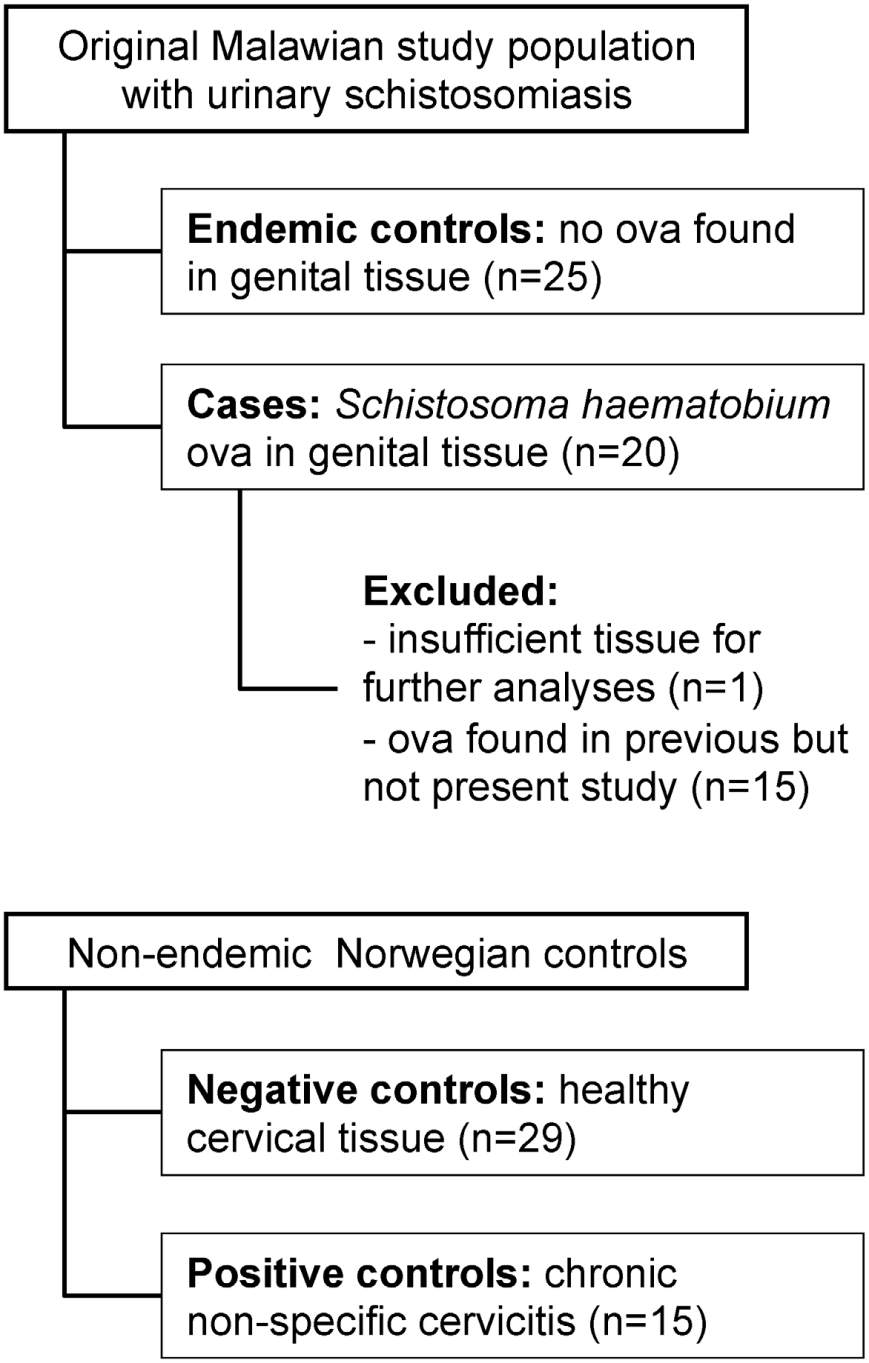
Overview of the study groups.

An overview of the study groups is given in Figure 1. The cases with *S. haematobium* ova in genital tissue included 17 cervical and three vaginal biopsies. The histopathological changes and ova viability were similar in the cervical and vaginal biopsies, and the biopsies were therefore analysed together. Malawian women with urinary schistosomiasis who were found not to have *S. haematobium* ova in the biopsy specimen served as endemic negative controls.

Non-endemic Norwegian control biopsies were selected by searching the database from 1998 for key word combinations of the anatomical site ‘cervix uteri’ and the morphological diagnoses ‘cervicitis’ and ‘normal morphology’. Women included in the negative non-endemic control group had histologically normal uterine ectocervices with intact tissue architecture. Excluded were endocervical biopsies and biopsies showing signs of pathology, i.e. more than 10 inflammatory cells per high-power field, non-specific vessel proliferation, epithelial hyperplasia, polyps, cysts, atypical or dysplastic changes, or signs of human papilloma virus (HPV) infection (i.e. clustered koilocytosis and dyskeratosis).

Women included in the positive non-endemic control group had chronic non-specific cervicitis; i.e. biopsies with a distinct, generalised infiltrate dominated by lymphocytes and/or plasma cells. Excluded were endocervical biopsies with more than 20 granulocytes or 5 eosinophils per high-power field and specimens with signs of HPV infection, erosion, ulcerations, granulation tissue or extra-vascular erythrocytes.

### Immunohistochemistry and histopathology

The biopsies were fixed in formalin, routinely processed, embedded in paraffin, sectioned and stained. Findings from the histological examination of haematoxylin and eosin (HE)-stained sections have been published previously [14].

For the analyses in this study, 3.5 µm thick serial sections of the included specimens were cut and placed on SuperFrost slides (Menzel-Gläser, Braunschweig, Germany) for HE-stain and on SuperFrost Plus slides (Menzel-Gläser) for immunohistochemical stains. The immunohistochemical stains were performed using Benchmark XT, Antibody diluent (251-018) and Detection Kit Ventana ultraView Universal DAB (760-500) (Ventana Medical Systems, Inc., Tucson, Arizona, USA), an automated immunostain system based on the ABC avidin-biotin-peroxidase method, including negative and positive controls. For identification of blood vessels, two endothelial cell markers were used; mouse monoclonal antibody CD31 (clone JC70A, Dako Denmark AS, Glostrup, Denmark) and rabbit polyclonal antibody von Willebrand Factor (vWF) (Ventana). All slides were examined microscopically for immunohistochemical antigen detection combined with histological identification.

### Microscopy and image analysis

The sections were examined using a Nikon Eclipse 80i microscope and photographed at 40× objective magnification, obtaining 1600 by 1200 pixels colour images using a SPOT Insight 2 Megapixel Firewire Color 3-shot digital camera attached to the microscope and a Hewlett-Packard Compaq stationary computer.

The morphological and immunohistochemical analyses were performed in a standardised manner. First, the HE-stained sections were evaluated. Ova were defined as ‘viable’ if miracidia with eosinophilic glands or germinal cells were identified [15], whereas ova containing dark purple stain identified histologically as calcification were defined as ‘calcified’. The histopathological tissue reaction was defined as ‘granulation tissue’ if dominated by neovascularisation with activation of endothelial cells and by immature fibroblasts. The tissue reaction was defined as ‘fibrosis’ if dominated by collagen rich stroma with scant mature fibroblasts.

A high-power field (40× objective magnification) of each HE-stained section containing *S. haematobium* ova was photographed with the ovum or ova located centrally. The area of tissue surrounding the ovum or ova in one such photograph was defined as ‘periovular’. In the controls, one high-power field of a subepithelial area was photographed, as this is the most common location for oviposition [15], [16]. This area was representative for the pathologist's overall diagnosis of the section. In order to analyse the same area in the consecutive immunohistochemically stained serial sections of each biopsy, the near-exact same areas, identified by histological anatomical structures, were photographed. In areas where the morphology of the immunohistochemically stained section differed from the HE-stained section, the closest approximation was photographed. All selections were controlled by an experienced pathologist (BR).

Immunostained blood vessels (by vWF and CD31) were counted in accordance with pre-established criteria [17], [18]. ‘Capillary buds’ were defined as vessel structures without an identifiable lumen or periendothelial structures, whereas all other stained vessel structures were counted as ‘established blood vessels’. The density of capillary buds and vessels were calculated per mm^2^. In sections with more than 300 capillary buds per mm^2^, the numbers were truncated to 300. Each photograph was counted manually and one in ten randomly selected photographs were quality controlled. Discrepancies were resolved by consensus, if necessary after consulting a second senior pathologist. Finally, each photograph was recounted.

### Statistical analyses

The statistical analyses and sample size estimation were performed with SPSS version 16.0 and PS Power and sample size calculations version 2.1.31. Most variables were not normally distributed and non-parametric tests were therefore used when studying associations. Medians and interquartile ranges were used to describe the results. The Mann-Whitney U and Kruskal-Wallis H tests were applied where appropriate. For the calculation of odds ratio (OR), it was necessary to tertilise the not normally distributed variables. Spearman's rank correlation coefficient was used when studying associations between continuous variables. Intra-observer variability was determined by calculating the intra-class correlation coefficient (ICC) after log-transformation of the data. A 5% significance level was used throughout.

## Results

The median age of the Malawian patients was 23.5 years (interquartile range (IQR) = 20.0–31.5), and the median age of the Norwegian control patients was 44.0 years (IQR = 30.5–55.5). In the biopsies with *S. haematobium* ova, the median number of ova per high-power field was 3 (IQR = 1–8), of which 80 per cent were calcified. Most ova were localised in the subepithelial tissue, whereas only a few ova were found deeper in the stroma. Calcified and viable ova were found together in the same biopsy in one patient only. No schistosome worms were seen.

**Figure 2:**
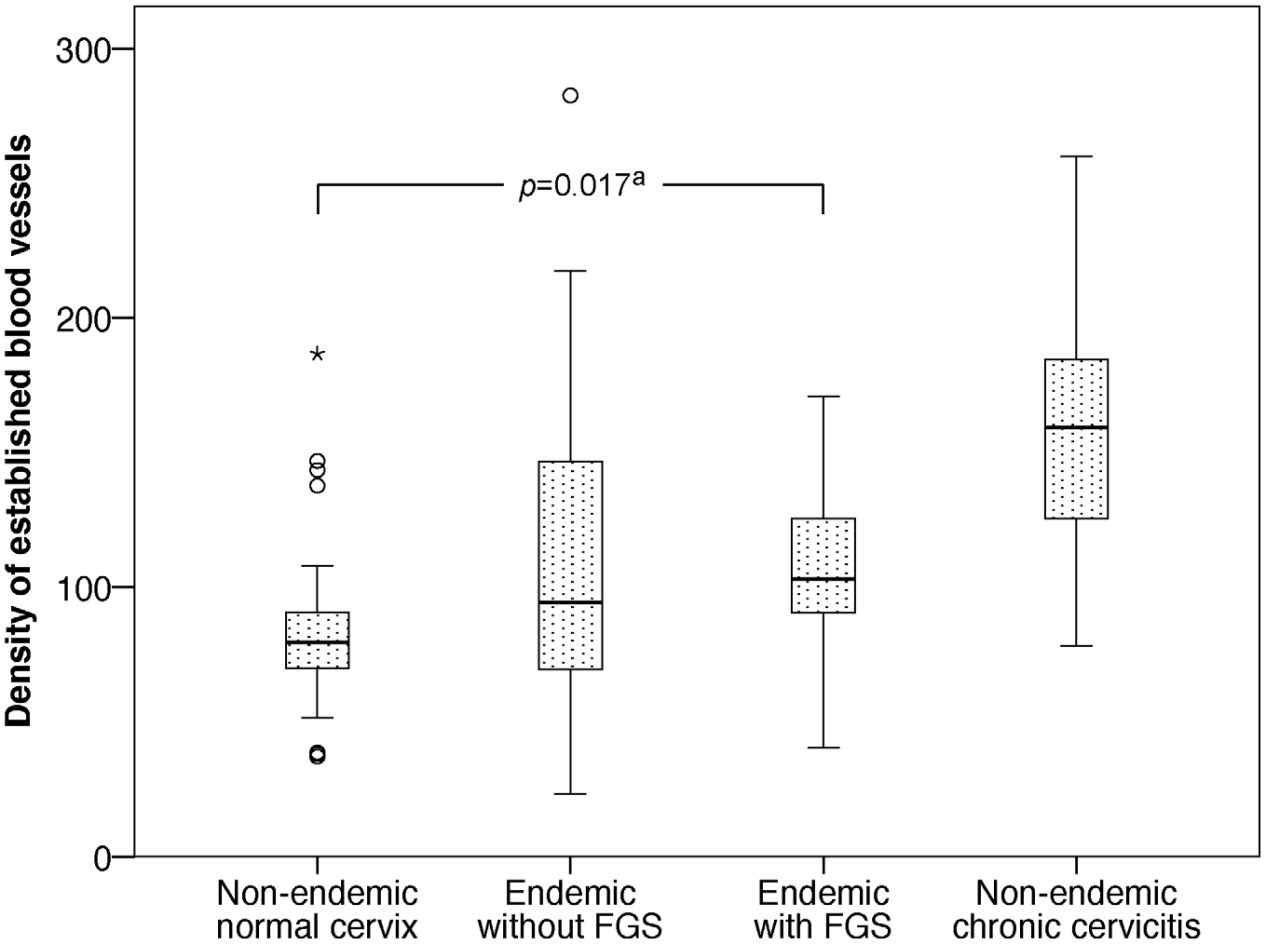
Density of established blood vessels per mm^2^ in the four study groups. Established blood vessels stained with von Willebrand Factor. FGS = cervicovaginal tissue with *S. haematobium* ova. ^a^Comparison of women with FGS and non-endemic women with normal cervical tissue.

**Figure 3:**
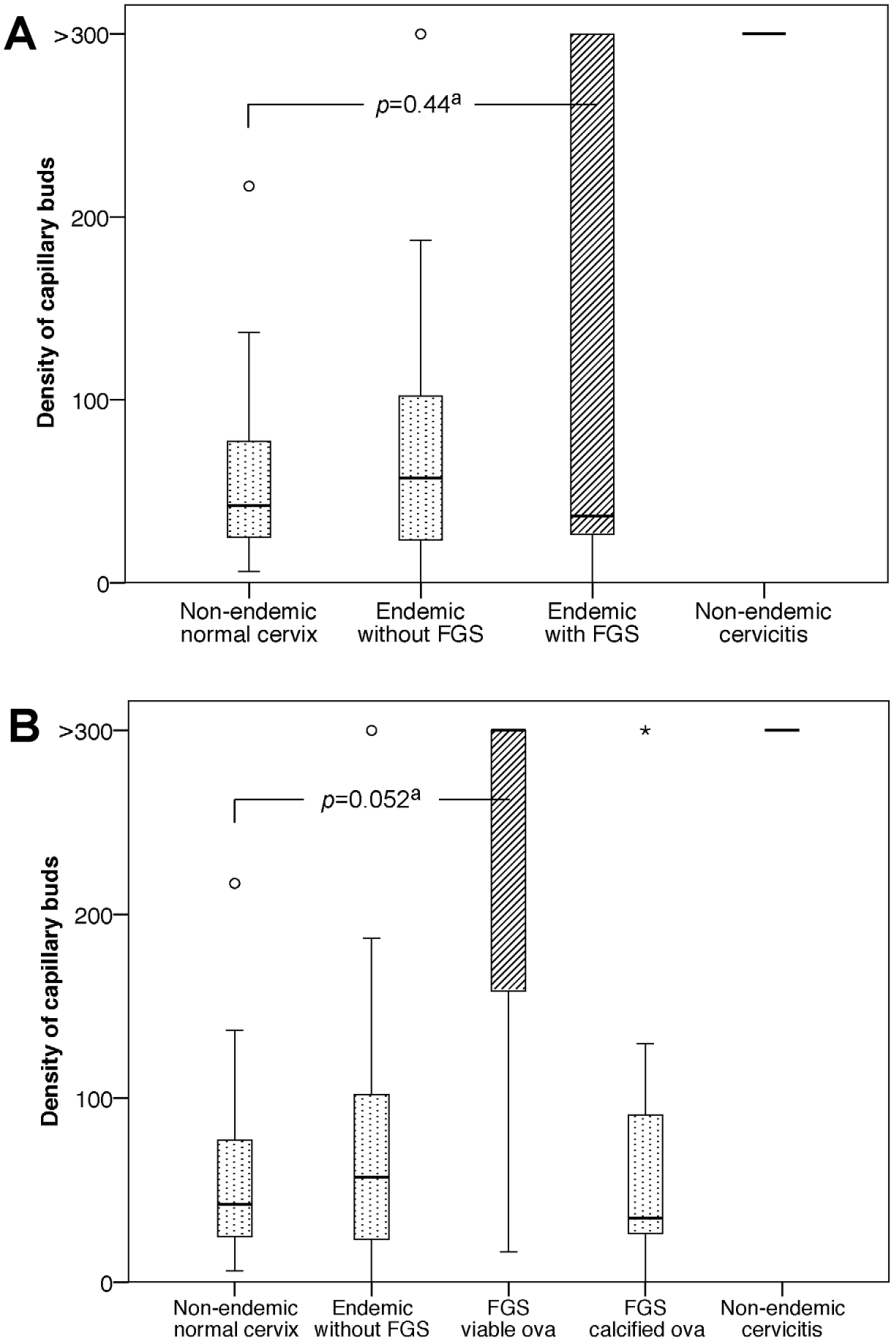
Density of capillary buds. **A. Density of capillary buds per mm^2^ in the four study groups.** Capillary buds stained with CD31; counts were truncated at 300 buds per mm^2^ for technical reasons. All non-endemic women with chronic cervicitis had capillary bud densities above 300 per mm^2^. FGS = cervicovaginal tissue with *S. haematobium* ova. ^a^Comparison of women with FGS and non-endemic women with normal cervical tissue. **B. Density of capillary buds per mm^2^ tissue with viable versus calcified ova.**

Figures 2 and 3A show the distributions of established blood vessels and capillary buds respectively per mm^2^ genital tissue in the four study groups. The periovular tissue contained significantly more established blood vessels compared with non-endemic healthy cervices (*p* = 0.017). However, ova viability was not significantly associated with the density of established blood vessels (*p* = 0.40). There was no significant difference in the density of established blood vessels between endemic biopsies with and without *S. haematobium* ova (*p* = 0.51).

As shown in Figure 3A, there were no significant differences in the density of capillary buds between the Malawian cases and the non-endemic or endemic negative controls (*p* = 0.44 and *p* = 0.95 respectively). However, there was a large variation in periovular capillary bud density. As shown in Figure 3B, there was a tendency towards a higher density of capillary buds surrounding viable ova compared with non-endemic healthy cervical tissue (OR = 13.0, Confidence Interval (CI) = 1.0–172.9, *p* = 0.052). Furthermore, viable ova were more often found in granulation tissue compared to calcified ova (*p* = 0.032). There was no association between calcified ova and fibrous tissue (*p* = 0.53). The main histopathological periovular tissue reactions are shown in Figures S1 and S2.

In the endemic cases with cervicovaginal *S. haematobium* ova, age was neither associated with the number of ova (*p* = 0.45) nor the viability status of the ova (*p* = 0.50). There was no significant difference in density of established blood vessels or capillary buds between tissue with viable ova and non-endemic tissue with chronic cervicitis (*p* = 0.20 and *p* = 0.053 respectively). All other study groups had significantly lower densities of established blood vessels and capillary buds than tissue with chronic cervicitis (data not shown).

The intra-observer reliability of counting established blood vessels and capillary buds were 0.93 (95% Confidence Interval (CI) = 0.90–0.95, p<0.001) and 0.88 (95% CI = 0.82–0.91, p<0.001), respectively.

## Discussion

To our knowledge, this is the first study to analyse the quantity and characteristics of the mucosal vasculature surrounding female genital *S. haematobium* ova. Similar to non-specific chronic cervicitis, the mucosa of the Malawian women diagnosed with genital schistosomiasis was significantly more vascularised than healthy cervical tissue of non-endemic controls. The denser vasculature consisted of established blood vessels as opposed to currently active neovascularisation. However, cases with viable ova contained granulation tissue rich in sprouting blood vessels significantly more often than cases with calcified ova.

Similar to previous reports of genital and urinary schistosomiasis, we found a variety of tissue reactions surrounding *S. haematobium* ova; ranging from marked periovular granulation tissue to fibrosis [14]–[16], [19]–[25]. Although fibrosis is regarded as the end-stage pathology in schistosome infected tissue [26], there was no significant association between calcified ova and fibrosis in our study.

This study has several limitations. Firstly, the sample size is small and the findings are prone to type 1 and 2 errors. Secondly, there may have been schistosome ova just outside the biopsy borders in presumed negative cases, which might explain why there was no significant difference in capillary density between endemic cases and controls in this study. As in most schistosomiasis studies, there was no true schistosomiasis negative endemic control group [27]. The differences between women with and without genital *S. haematobium* ova may therefore have been underestimated. Thirdly, the analyses may have been affected by the age of the endemic biopsies, and by endothelial cell activation or vWF which may be elevated in HIV positive individuals [28]. The HIV prevalence has subsequently been estimated to be approximately 10 per cent of the population in 1994 [2], but individual HIV diagnosis was not a part of the original project and could therefore not be done. Lastly, although the specimens were evaluated several times by two investigators who attempted to be objective, blinding was not possible due to the presence of schistosome ova in the tissue.

Previous studies on blood vessel proliferation and schistosomiasis have suggested that soluble egg antigens (SEA) from viable *S. mansoni* ova induce neovascularisation by stimulating endothelial cell activation and proliferation [10], [11]. Recent experimental studies indicate that blood vessel proliferation in *S. mansoni* not only accompanies hepatic fibrogenesis, but possibly also regression of fibrosis after treatment [29]. To our knowledge, there have been no studies on *S. haematobium* and blood vessel proliferation.

Female genital schistosomiasis is associated with genital mucosal bleeding tendency; a clinical finding that might support the suggested HIV susceptibility in these women [30]. Endothelial cell surface proteoglycans have been reported to serve as HIV-1 receptors, and endothelial cells have even been suggested to harbour HIV [31]–[34]. Finally, an increase in microvessel density may impair the original tissue structure and lead to easy disruption of the genital mucosal barrier [35], [36].

Women living in endemic areas are prone to high burdens of infection and frequent reinfections with *S. haematobium* [37]. The histopathological correlates of the clinical signs in female genital schistosomiasis, e.g. abnormal blood vessel proliferation and contact bleeding, have not yet been studied in detail [38]. It is therefore not known which clinical manifestations may increase the risk of reproductive tract morbidity or possibly HIV acquisition. Hence it is not yet possible to give clinical advice related to the risks and consequences of the typical lesions in female genital schistosomiasis. In order to determine the implications for the patients, further studies are needed to correlate the clinical signs to morphological and immunohistochemical findings.

In conclusion, this study shows that female genital mucosa infected with *S. haematobium* is significantly more vascularised than healthy cervical tissue. Viable schistosome ova are more often surrounded by highly vascularised granulation tissue compared with calcified ova. These results might contribute to improve the understanding of the pathophysiological mechanisms in female genital schistosomiasis and of the postulated association with HIV infection. However, further studies are needed to validate these findings, to study the immunological tissue reaction and to explore the clinical correlates of the various histopathological manifestations in female genital schistosomiasis.

## Supporting Information

Figure S1 **Section of uterine cervix with calcified** ***S. haematobium*** **ova. A. Histopathology** Calcified schistosome ova (white arrows) with periovular collagenised fibrous tissue (long, thin black arrows), scant mature fibroblasts (short, thin black arrows) and established blood vessels (thick black arrow). Haematoxylin and eosin (HE), 40× objective magnification. **B. Immunohistochemical detection of established mucosal blood vessels** Calcified schistosome ova (white arrows) with periovular established blood vessels (black arrows). von Willebrand Factor, 40× objective magnification.
(TIF)

Figure S2 **Section of vagina with viable** ***S. haematobium*** **ova. A. Histopathology** Viable schistosome ova and shell fragments (white arrows) surrounded by giant cells (long, thin black arrows), granulation tissue, i.e. endothelial cell proliferation and activation (E) and proliferation of immature fibroblasts (short, thin black arrows), and inflammation with marked eosinophilia (Eos). A tissue artifact (Art) traverses the lower half of the image. Haematoxylin and eosin (HE), 40× objective magnification. **B. Immunohistochemical detection of mucosal blood vessel budding** Viable schistosome ova (white arrows) with abundant periovular capillary blood vessel budding (black arrows). CD31, 40× objective magnification.
(TIF)
