# Supplementary figures and images for: Increased Vascularity in Cervicovaginal Mucosa with Schistosoma haematobium Infection
Source: PLoS Negl Trop Dis. 2011 Jun 7;5(6):e1170. doi: 10.1371/journal.pntd.0001170 (PMC3110160; doi:10.1371/journal.pntd.0001170)

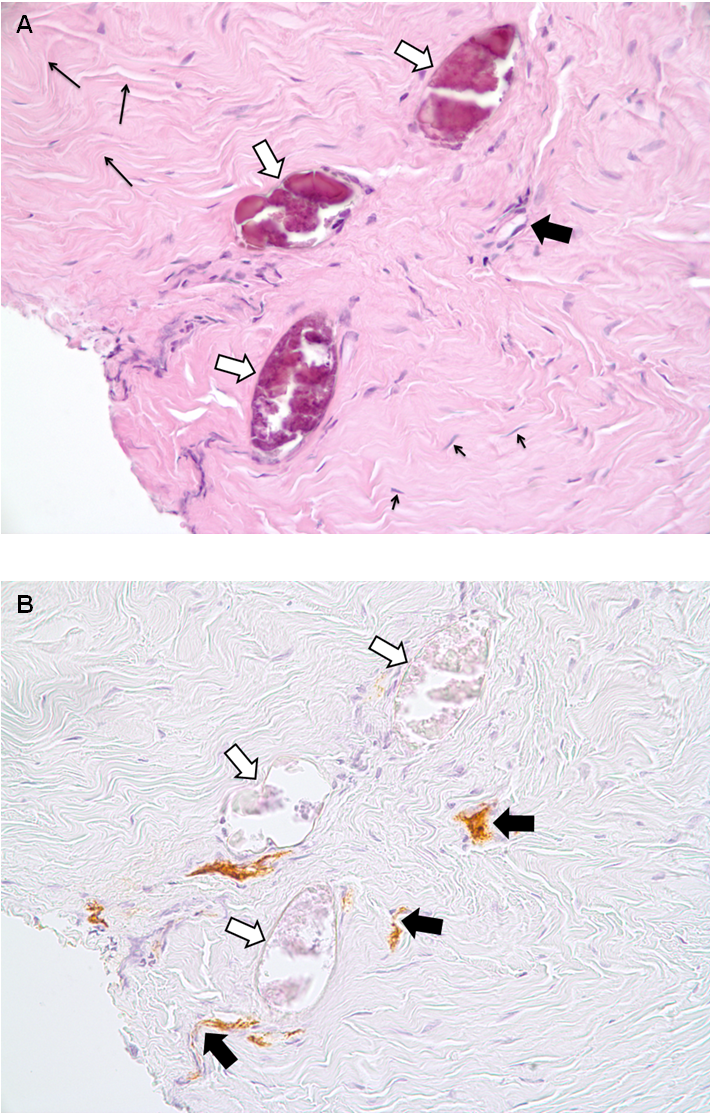

Supplement: Figure S1 — Section of uterine cervix with calcified S. haematobium ova. A. Histopathology Calcified schistosome ova (white arrows) with periovular collagenised fibrous tissue (long, thin black arrows), scant mature fibroblasts (short, thin black arrows) and established blood vessels (thick black arrow). Haematoxylin and eosin (HE), 40× objective magnification. B. Immunohistochemical detection of established mucosal blood vessels Calcified schistosome ova (white arrows) with periovular established blood vessels (black arrows). von Willebrand Factor, 40× objective magnification. (TIF) [file pntd.0001170.s001.tif]

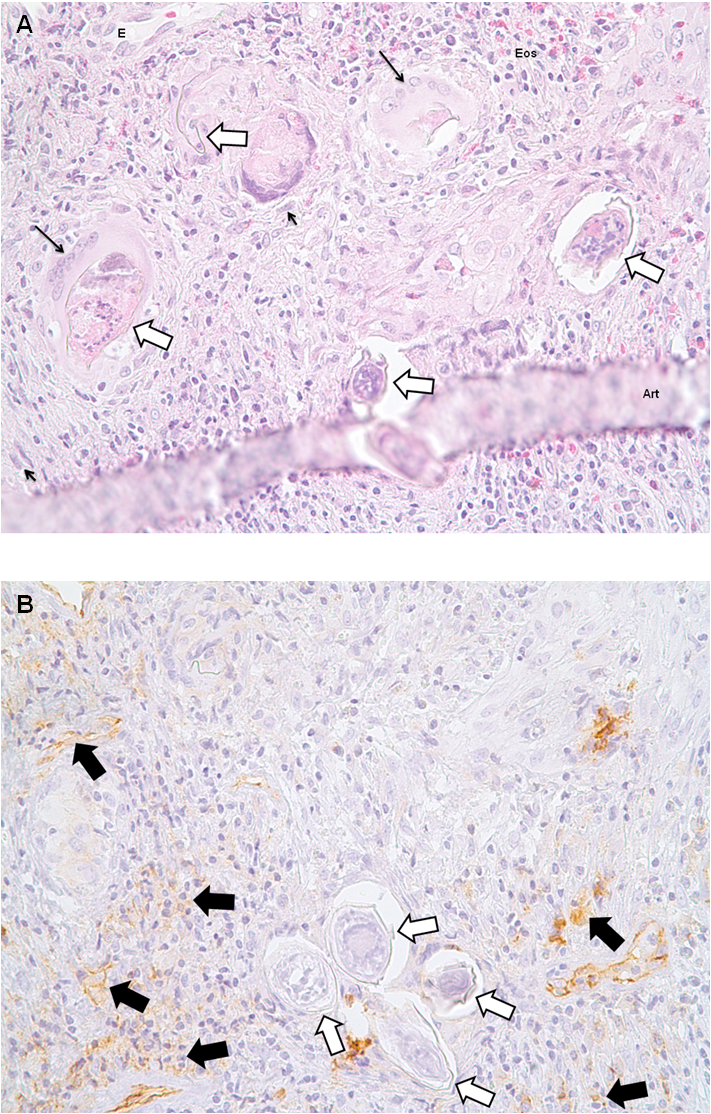

Supplement: Figure S2 — Section of vagina with viable S. haematobium ova. A. Histopathology Viable schistosome ova and shell fragments (white arrows) surrounded by giant cells (long, thin black arrows), granulation tissue, i.e. endothelial cell proliferation and activation (E) and proliferation of immature fibroblasts (short, thin black arrows), and inflammation with marked eosinophilia (Eos). A tissue artifact (Art) traverses the lower half of the image. Haematoxylin and eosin (HE), 40× objective magnification. B. Immunohistochemical detection of mucosal blood vessel budding Viable schistosome ova (white arrows) with abundant periovular capillary blood vessel budding (black arrows). CD31, 40× objective magnification. (TIF) [file pntd.0001170.s002.tif]
